# Long-term effect of COVID-19 infection on hemodialysis patients: Should we follow hemodialysis patients more closely?

**DOI:** 10.1093/ckj/sfab265

**Published:** 2021-12-09

**Authors:** Atalay Demiray, Asiye Kanbay, Mehmet Kanbay

**Affiliations:** Department of Medicine, Koc University School of Medicine, Istanbul, Turkey; Department of Pulmonary Medicine, Istanbul Medicana Atasehir Hospital, Istanbul, Turkey; Department of Medicine, Division of Nephrology, Koc University School of Medicine, Istanbul, Turkey

**Keywords:** anti-SARS-CoV-2 antibodies, COVID-19, hemodialysis, mortality, outcomes

## Abstract

During the coronavirus disease 2019 (COVID-19) pandemic, hemodialysis patients constitute one of the most vulnerable patient populations as they have more significant comorbidities and need to visit healthcare settings frequently even under pandemic conditions. It was also largely demonstrated that hemodialysis patients have high mortality rates with severe to fatal disease due to COVID-19 during their initial hospitalization. Even though the functional decline and fatigue after severe infections are not a novel entity, some long-term effects of COVID-19 have drawn attention with their prolonged effects even after discharge. A recent prospective, observational study by Carriazo *et al.* provided the first evidence to compare long-term mortality rates of hemodialysis patients with and without COVID-19. Carriazo *et al.* stated a hazard ratio of 3.00 for the mortality rates of hemodialysis patients over a 1-year follow-up period after their COVID-19 diagnosis. They emphasized that the high mortality rates of hemodialysis patients with COVID-19 are not limited to the initial hospitalization period but also continue after discharge, especially in the first 3 months. In light of this study, it can be recommended that hemodialysis patients with COVID-19 should be monitored closely and continuously, and hemodialysis patients should be prioritized for vaccination against COVID-19 with close follow-up for their antibody levels.

Carriazo *et al.* [1] conducted a prospective, observational study to analyze the serological and clinical outcomes of 56 hemodialysis patients with coronavirus disease 2019 (COVID-19) for a year after their diagnosis. While current literature has reported COVID-19-related mortality among hemodialysis patients during the initial hospitalization or 1-month mortality after the diagnosis, this unique paper reported the outcomes of hemodialysis patients with prolonged follow-up. Carriazo *et al.* [1] showed that high mortality rates of COVID-19 in hemodialysis patients are not limited to the initial hospitalization but also continue in the first year after the diagnosis. Among 56 hemodialysis patients with COVID-19, 20 (35.7%) died over 12 months, with only 6 (11%) of them dying during the initial admission. Among 144 hemodialysis patients without COVID-19 infection, 21 (14.6%) died over 12 months. After 1 year of follow-up, they reported a hazard ratio (HR) for death for COVID-19 patients of 3.00 (1.62–5.53) with a log-rank P = 0.00023. The findings of Carriazo *et al*. made us question our approach to monitoring hemodialysis patients after COVID-19 and the possible prolonged effects of COVID-19 on hemodialysis patients (Figure 1).

**FIGURE 1:**
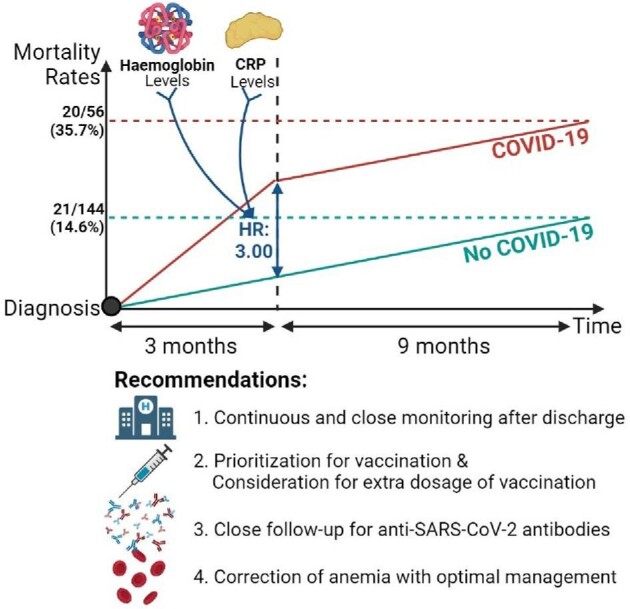
The simplified graph for mortality rates of hemodialysis patients with and without COVID-19 and recommendations for hemodialysis patients with COVID-19. CRP, C-reactive protein.

The number of patients with end-stage kidney disease is rising with the aging population, and hemodialysis patients constitute a bigger burden in nephrology clinics [2, 3]. The management and follow-up of hemodialysis patients have been a particular challenge for nephrologists during the COVID-19 pandemic [4]. COVID-19 is a highly infectious viral disease with a reported case fatality rate of 15% in hemodialysis patients [5]. Even though several drug regimens and protocols for COVID-19 have been prepared and worldwide vaccination against COVID-19 continues, the management of hemodialysis patients with COVID-19 appeared more challenging than other patient populations due to some unknown aspects of COVID-19 [6]. In light of the present study, we can speculate two challenging aspects of COVID-19 infection that need to be elucidated are post-COVID-19 syndrome and the rapidly declining nature of severe acute respiratory syndrome coronavirus 2 (SARS-CoV-2) antibodies in hemodialysis patients.

Patients with chronic kidney disease (CKD), especially hemodialysis patients, have several risk factors and comorbidities associated with severe or fatal COVID-19 [7–9]. Hemodialysis patients are also vulnerable to COVID-19 because of impaired immune function and frequent hospital visits as part of their life-sustaining therapy [9]. A recent study with 576 hemodialysis patients showed in-hospital mortality (16.3%) due to COVID-19 and its determinants during hospitalization [10]. However, it was unknown whether the effects of COVID-19 and the high mortality rate were presented only acutely and limited to hospitalization or not. The study of Carriazo *et al.* [1] demonstrated that high mortality rates of COVID-19 and its effects on hemodialysis patients could continue for 1 year, especially in the first 3 months after the diagnosis.

After COVID-19 discharge, functional limitation over a long period is very common due to post-infection fatigue. It is difficult to distinguish this functional limitation and the rehabilitation phase after discharge from the post-COVID syndrome. However, several studies demonstrated prolonged effects of COVID-19, and some indicated post-COVID-19 syndrome [11]. Even though the long-term effects of COVID-19 on CKD patients have not been clearly demonstrated, the need for special rehabilitation programs and monitoring after discharge is recommended [12]. In light of the current study, we can speculate that the long-term consequences of COVID-19 on hemodialysis patients may not be just temporary post-infection fatigue, but a more severe entity that needs to be elucidated to explain significantly high mortality rates even after discharge.

The second important issue of COVID-19 for hemodialysis patients is the rapid decline of SARS-CoV-2 antibodies compared with the general population. It is not novel that hemodialysis patients receiving immunosuppression therapies have suppressed immune responses against infections and vaccinations. Additionally, immune responses and antibody formation in hemodialysis patients not on immunosuppressants were lower than in the general population, so they are more prone to re-infection and faster loss of protection following vaccination. According to the study by Carriazo *et al.*, only 30.6% of all COVID-19 survivor hemodialysis patients had anti-SARS-CoV-2 IgG after the 1-year follow-up. A decline in antibody levels against COVID-19 was observed between 6 and 12 months, while some patients experienced re-infection and some never developed positive antibodies even after the vaccination.

Besides comorbidities and altered immune responses, hemodialysis patients need to visit hemodialysis centers for their life-saving treatment three times a week, and each visit can be a potential source for the transmission of COVID-19. Because these concerning aspects are specific to hemodialysis patients, it was broadly suggested that hemodialysis patients should be prioritized for vaccination and need to receive their COVID-19 vaccine as soon as possible [13]. Hemodialysis patients should be followed more closely for their immune status, serological response and possible re-infection. The study of Carriazo *et al.* [1] pointed out that the presence of anti-SARS-CoV-2 immunoglobulin G in hemodialysis patients decreased from 36/49 (73.4%) initially to 27/44 (61.3%) at 6 months and 14/36 (38.8%) at 12 months. These findings made us question the immunity and antibody protection of hemodialysis patients with COVID-19 infection or vaccination. Therefore, possible COVID-19 re-infection should be kept in mind for hemodialysis patients even following vaccination.

There are some limitations of the study that could be addressed in the future. Carriazo *et al.* study presented a single-center experience with a limited sample size. Multi-center data with bigger samples could validate and enlighten more about the long-term consequences of COVID-19 on hemodialysis patients. Even though the study presented clear data indicating high mortality rates even after initial hospitalization, more associations such as re-infections, hospitalizations due to other causes with longer follow-up periods should be assessed better for differentiating post-COVID syndrome (or effects) from general post-infection fatigue (or functional limitations).

In short, COVID-19 seems to have long-term consequences on hemodialysis patients with higher mortality rates not limited to the initial hospitalization period but also 1 year after the diagnosis. All infections tend to present more severely in hemodialysis patients due to comorbidities, drug regimens and vulnerability than in other patient populations, and COVID-19 is not an exception. COVID-19 is not the first pandemic, and it will not be the last one. Future pandemics will also be threats to the well-being of hemodialysis patients. It should be kept in mind that hemodialysis patients always constitute a more vulnerable population in pandemics, epidemics and infectious disease in general. Considering an increasing number of hemodialysis patients worldwide, their management in the case of COVID-19 and possible future infections should be well-tailored and closely followed.
